# Eight-Year Surveillance of *Candida albicans* Bloodstream Infections in an ICU in Turkey

**DOI:** 10.22034/cmm.2025.345430.1676

**Published:** 2026-02-01

**Authors:** Süleyha Hilmioğlu-Polat, Dilek Yeşim-Metin, Melike Yaşar, Aylin Döğen, Ayşe Serin, Mehmet Sami Serin, Ramazan Gümral, Mohsen Seyedmousavi, Haleh Rafati, Somayeh Sharifynia, Yalda Tamaddon, Ali Tolooe, Ayatollah Nasrollahi Omran, Macit Ilkit, Corné H.W. Klaassen, Amir Syedmousavi

**Affiliations:** 1 Division of Mycology, Department of Microbiology, Faculty of Medicine, University of Ege, Izmir, Turkey; 2 Department of Pharmaceutical Microbiology, Faculty of Pharmacy, University of Mersin, Mersin, Turkey; 3 Department of Forensic Medicine, Faculty of Medicine, University of Cukurova, Adana, Turkey; 4 Department of Microbiology, Gulhane Training and Research Hospital, University of Health Sciences, Ankara, Turkey; 5 Center of Expertise in Microbiology, Infection Biology and Antimicrobial Pharmacology, Tehran, Iran; 6 Clinical Tuberculosis and Epidemiology Research Center, National Research Institute of Tuberculosis and Lung Diseases, Shahid Beheshti University of Medical Sciences, Tehran, Iran; 7 Department of Small Animal Internal Medicine, Faculty of Specialized Veterinary Sciences, Science and Research Branch, Islamic Azad University (IAU), Tehran, Iran; 8 Department of Pathobiology, Ontario Veterinary College, University of Guelph, Guelph, Ontario, Canada; 9 Department of Mycology, Faculty of Medical Sciences, Tonekabon Branch, Islamic Azad University, Tonekabon, Iran; 10 Division of Mycology, Department of Microbiology, Faculty of Medicine, University of Çukurova, Adana, Turkey; 11 Department of Medical Microbiology and Infectious Diseases, Erasmus−MC University Medical Center, Rotterdam, The Netherlands; 12 Microbiology Service, Department of Laboratory Medicine, Clinical Center, National Institutes of Health, Bethesda, MD, United States of America

**Keywords:** Antifungal susceptibility, *Candida albicans*, Candidemia, microsatellite genotyping, Turkey

## Abstract

**Background and Purpose:**

Although the distribution of *Candida* species has shifted over the past three decades, *Candida albicans* remains the leading cause of nosocomial bloodstream infections.

**Materials and Methods:**

This study examined the genetic diversity and antifungal susceptibility of 174 independent *C. albicans* bloodstream isolates obtained from intensive care unit patients in Turkey. Microsatellite analysis across five loci identified 21 allelic genotypes for elongation factor 3 (*CEF3*), 12 for carbonic anhydrase III (*CAIII*), 11 for LOC4, 11 for zinc finger transcription factor-1 (*ZNF1*), and 9 for killer toxin-resistance protein-6 (KRE6). The multiplex analysis yielded 104 different multi-loci allelic combinations for the 174 strains analyzed, resulting in a discriminatory power of 0.98.

**Results:**

Locus-by-locus microsatellite analysis revealed that certain *C. albicans* strains from the intensive care unit shared identical genotypes. The geometric mean minimum inhibitory concentrations of antifungal agents, in ascending order, were 0.02 mg/L for itraconazole, voriconazole, and posaconazole, 0.03 mg/L for anidulafungin, 0.13 mg/L for caspofungin, 0.20 mg/L for flucytosine, 0.62 mg/L for fluconazole, and 0.67 mg/L for amphotericin B.

**Conclusion:**

Findings showed considerable genetic diversity among bloodstream *C. albicans* isolates, while antifungal resistance remained uncommon. No significant association was observed between antifungal susceptibility and the genotypes analyzed. The presence of identical genotypes among certain strains points to an endogenous origin and a potential common source of infection within the hospital, warranting further investigation.

## Introduction

*Candida* species represent the most common cause of healthcare-associated fungal bloodstream infections globally [1, 2], and the global incidence of candidemia is rising along with the growing complexity of surgical procedures, expanding population of patients at high risk of infection, and changes in patient demographics [2]. Moreover, candidemia increases patient mortality risk and contributes to longer hospital stays and higher healthcare costs [3, 4]. Despite shifts in species distribution over the past three decades, *C. albicans* continues to be the most frequently isolated species in candidemia, followed by *C. glabrata*, *C. parapsilosis*, *C. tropicalis*, and *C. krusei* [4]. However, the list of reported non-albicans Candida species continues to grow, with *Candidozyma* (*Candida*) *auris* recently reported as a rapidly emerging cause of hospital-acquired multidrug-resistant infections worldwide [5]. Given that delays in appropriate antifungal therapy are associated with increased mortality, antifungal-susceptibility testing of *Candida* plays a useful role in managing *Candida* infections [6]. Identification of the molecular genotypes of corresponding *Candida* species contributes to a better understanding of the origin and mode of transmission of nosocomial infections [7]. These findings can help identify endemic genotypes circulating within hospitals [8].

In a previous study performed at a university hospital in Izmir, *C. albicans* was the most frequently isolated *Candida* species from blood cultures, accounting for 43.8% of cases [9]. However, there are limited data regarding the antifungal-resistance and molecular diversity of *C. albicans* bloodstream strains in Turkey [10, 11]. Therefore, this study aimed to examine the antifungal susceptibility and microsatellite genotypes of a large collection of bloodstream *C. albicans* isolates obtained from patients in Turkey over the past eight years.

## Materials and Methods

### Fungal isolates

This study analyzed 174 independent bloodstream *C. albicans* isolates obtained between 2006 and 2014 from Ege University Hospital, İzmir, Turkey, including only one isolate per patient. All samples were initially identified based on conventional morphology using CHROMagar *Candida* (Becton Dickinson, Heidelberg, Germany), chlamydospore formation in cornmeal-80 agar (Difco, Detroit, MI, USA), and carbohydrate assimilation using the API 20C AUX system (bioMérieux, Marcy l’Étoile, France). The identities of all isolates were then confirmed by polymerase chain reaction (PCR) for the hyphal wall protein 1 (*HWP1*) gene, as described previously [12]. *Candida albicans* ATCC 90028 (ATCC, Manassas, VA, USA) was used as a positive control in all experiments.

### DNA Isolation

The DNA was isolated as described previously [11]. Briefly, yeast cells were cultured in Sabouraud’s broth at 30 °C, and a yeast suspension was prepared by adding 400 µL of Tris-EDTA-NaCl buffer (10 mM Tris/HCl at pH 8.0, 5 mM EDTA, and 0.5% NaCl), 10 µL of lyticase (2 g), and 100 µL of 10% sodium dodecyl sulfate, followed by vortexing. This suspension was incubated overnight in a water bath at 56 ºC, resuspended in 600 µL of phenol:chloroform:isoamyl alcohol (25:24:1, v/v), and pelleted by centrifugation at 12,000 × g for 5 min, followed by transfer of the supernatant to a sterile tube.

The supernatant was then mixed with 600 µL of isopropyl alcohol, corresponding to approximately twice the volume of the supernatant, centrifuged at 12,000 × g for 10 min, followed by discarding the supernatant and washing the pellet in 1 mL of cold 70% ethanol before centrifugation at 12,000 × g for 10 min. The resulting supernatant was carefully discarded, and the tubes were placed upside-down on clean paper towels to dry for 10 min. The DNA was suspended in 100 µL of sterile double-distilled water (ddH_2_O) and stored at −20 ºC prior to PCR amplification.

### Microsatellite polymerase chain reaction amplification

Microsatellite genotyping of all *C. albicans* isolates was performed using five short-nucleotide-repeat loci [*CAIII*, *CEF3*, *KRE6*, *LOC4*, and *ZNF1*] with fluorescence-labeled primers, as previously described [11]. Multiplex PCR for *CAIII*, *LOC4*, *ZNF1*, and *KRE6* was performed in 10-L reaction volumes containing 5 µL of 2× multiplex PCR reagent (Qiagen, Hilden, Germany), 1 µL of each primer (1g/l; forward and reverse), 3 µL of ddH_2_O, and 1 µL of genomic DNA. Ampliﬁcations were carried out in a thermocycler (TC_Pro; Boeckel and Co., Hamburg, Germany) using the following program: initial denaturation at 95 ºC for 15 min, 35 cycles at 94 ºC for 30 s, at 57 ºC for 90 s, and at 72 ºC for 60 s, followed by a final extension at 72 ºC for 10 min. A separate PCR was performed for *CEF3* in 25-L reaction volumes containing 1 × PCR buffer (20 mM Tris hydrochloride (pH 8.4) and 50 mM potassium chloride), 0.5 mM of each deoxynucleoside triphosphate (Fermentas, Waltham, MA, USA), 1 µL of each primer (for hexachloro-fluorescein-labeled *CEF*), 1.5 mM MgCl_2_ (Fermentas), 3 µL genomic DNA, and 1.25 U Taq DNA polymerase (New England BioLabs, Hitchin, UK). The PCR ampliﬁcations for *CEF3* comprised an initial denaturing step at 95 ºC for 5 min, followed by 40 cycles at 95 ºC for 30 s, at 56 ºC for 30 s, and at 72 ºC for 45 s, and a ﬁnal extension step at 72 ºC for 5 min. Genomic DNA from *C. albicans* ATCC 18804 (CBS 562) was used as a positive control to test the accuracy of the typing assay. The PCR products were run on an ABI 3130 Genetic Analyzer (Applied Biosystems, USA) together with the GeneScan-LIZ 500 size standard (Applied Biosystems). Fragment sizes were determined using the GeneScan software (version 3.2).

### In vitro antifungal-susceptibility testing

Isolates were tested for *in vitro* susceptibility to fluconazole (Pfizer Central Research, Sandwich, UK), itraconazole (Janssen Research Foundation, Beerse, Belgium), posaconazole (Merck, Whitehouse Station, NJ, USA), voriconazole (Pfizer Central Research, Sandwich, UK), amphotericin B (Bristol-Myers Squibb, Woerden, The Netherlands), flucytosine (ICN Pharmaceuticals, Zoetermeer, The Netherlands), caspofungin (Merck Sharp and Dohme BV, Haarlem, The Netherlands), and anidulafungin (Pfizer Central Research) using the European Committee on Antimicrobial Susceptibility Testing broth microdilution method for yeasts [13].

Final concentrations of the antifungal agents ranged from 0.016 to 16 mg/L for amphotericin B, itraconazole, voriconazole, posaconazole, anidulafungin, and caspofungin, and from 0.06 to 64 mg/L for fluconazole and 5-flucytosine.

Results were analyzed using a microtitration plate spectrophotometric reader (Anthos htIII; Anthos Labtec Instruments, Salzburg, Austria). *Candida parapsilosis* (ATCC 22019) and *C. krusei* (ATCC 6258) were included as quality controls in all experiments. Each strain was tested in three independent replicates on separate days.

### Statistical analysis

Data were analyzed using GraphPad Prism version 7.0 for Mac (GraphPad Software, San Diego, CA, USA). Genotypic diversity and minimum inhibitory concentration (MIC) distributions among isolates from different locations were compared using the student’s t test and Mann-Whitney-Wilcoxon test, with *p* ≤ 0.05 (two-tailed) considered statistically significant. Allele and genotype frequencies were calculated using ARLEQUIN software (version 3.5) [14], and the combined discriminatory power (DP) of the markers was determined as described by Hunter and Gaston [15].

## Results

The PCR amplification of the *HWP1* gene in all 174 *C. albicans* isolates produced a DNA fragment of ~941 bp. No isolated yielded fragments corresponding to *C. dubliniensis* (569 bp), *C. africana* (740 bp), or *C. stellatoidea* (800 bp).

The geometric mean MICs for all isolates, in ascending order, were 0.02 mg/L for itraconazole, voriconazole, and posaconazole, 0.03 mg/L for anidulafungin, 0.13 mg/L for caspofungin, 0.20 mg/L for flucytosine, 0.62 mg/L for fluconazole, and 0.67 mg/L for amphotericin B.

As shown in Table 1, itraconazole, voriconazole, and posaconazole showed the highest *in vitro* activity against all strains, with MICs ranging from 0.016 mg/L to 0.031 mg/L. The widest MIC ranges were measured for flucytosine (0.063–4 mg/L) and fluconazole (0.063–16 mg/L).

**Table 1:** Geometric means of the MICs, MIC ranges, and the MIC_50_ and MIC_90_ values obtained by testing the susceptibility of 174 bloodstream *Candida albicans* strains to eight antifungal agents, according to the European Committee on Antimicrobial Susceptibility Testing method.

	Amphotericin B	Flucytosine	Fluconazole	Itraconazole	Voriconazole	Posaconazole	Anidulafungin	Caspofungin
Geometric mean	0.67	0.20	0.62	0.02	0.02	0.02	0.030	0.13
Range	0.5-1	0.063–4	0.062–16	0.016–0.031	0.016	0.016	0.016–1	0.031–1
MIC_50_	0.5	0.125	0.25	0.016	0.016	0.016	0.016	0.125
1MIC_90_	1	0.25	0.5	0.016	0.016	0.016	0.016	0.125

MIC: minimum inhibitory concentration; MIC_50_: 50% minimum inhibitory concentration; MIC_90_: 90% minimum inhibitory concentration.

One isolate was resistant to flucytosine, and one isolate to fluconazole. The MIC range of the echinocandin drugs was 0.016-1 mg/L for anidulafungin and 0.031-1 mg/L for caspofungin. All *C. albicans* isolates except one were susceptible to both echinocandins.

Genotyping of five microsatellite loci revealed considerable diversity among the 174 *C. albicans* strains. Based on the different allelic combinations, 21 genotypes were identified for *CEF3* (DP = 0.88), 12 for *CAIII* (DP = 0.71), 11 for *LOC4* (DP = 0.71), 11 for *ZNF1* (DP = 0.78), and 9 for *KRE6* (DP = 0.74). The most common genotypes were 99–99 (genotype 4; 50.6%) for *CAIII*, 121–121 (genotype 8; 49.4%) for *LOC4*, 135–135 (genotype 1; 39.1%) for *KRE6*, 168–168 (genotype 2; 35.1%) for *ZNF1*, and 135–145 (genotype 18; 25.3%) for *CEF3* (Table 2). The multiplex analysis yielded 104 different multi-loci allelic combinations for the 174 strains analyzed, resulting in a DP of 0.98 (Tables 2 and 3).

**Table 2:** Characteristics of the five microsatellite loci

Locus	No. of alleles	Size range (bp)	Allele frequency	No. of genotypes	Obs-Het %	DP
CEF3	15	119-153	0.003-0.356	21	79.3	0.88
*CAIII*	6	96-112	0.020-0.667	12	42.5	0.71
*KRE6*	5	135-145	0.003-0.517	9	37.4	0.74
*LOC4*	6	115-130	0.170-0.572	11	37.9	0.71
*ZNF1*	7	162-188	0.003-0.592	11	52.9	0.78
Total				104		0.98

DP: discrimination power, bp: base pair, Obs-Het: observed heterozygosity.

**Table 3:** The 104 different genotypes were observed among 174 independent bloodstream *Candida albicans* isolates.

code	Isolate no.	*CaIII*	*LOC4*	*CEFIII*	*KRE6*	*ZNF*	No. of patients
1	18	96-96	121-121	120-120	138-145	168-174	1
2	19	96-96	121-121	144-144	138-145	168-168	1
3	38	96-99	119-119	135-138	138-138	180-180	1
4	4	96-99	119-121	135-138	135-135	168-168	1
5	4	96-99	119-130	131-135	135-145	168-168	1
6	130	96-99	121-121	124-134	138-145	168-168	1
7	118	96-99	121-121	129-135	138-138	168-174	1
8	6, 7	96-99	121-121	129-135	138-144	168-174	2
9	76	96-99	121-121	129-135	138-145	168-174	1
10	84	96-99	121-121	129-135	138-145	168-188	1
11	121	96-99	121-121	135-135	138-145	180-180	1
12	45	96-107	118-121	134-145	135-138	168-168	1
13	35, 111	96-107	130-130	135-135	135-138	168-174	2
14	148	99-99	115-121	134-135	138-144	168-168	1
15	32	99-99	115-121	134-145	135-135	174-174	1
16	101	99-99	118-118	129-144	138-138	168-168	1
17	1	99-99	118-118	131-135	138-138	180-180	1
18	36	99-99	118-118	135-145	138-138	168-174	1
19	105	99-99	118-121	124-134	135-135	168-168	1
20	152	99-99	118-121	129-134	135-138	168-168	1
21	47	99-99	118-121	135-138	135-138	171-171	1
22	150	99-99	118-121	135-145	135-138	168-168	1
23	167	99-99	118-121	135-145	135-138	174-174	1
24	65	99-99	118-130	135-144	138-138	168-168	1
25	31, 57, 90	99-99	118-130	135-144	138-138	168-180	3
26	169	99-99	119-119	135-145	135-138	168-180	1
27	25	99-99	119-130	119-129	138-138	168-168	1
28	14	99-99	121-121	120-120	135-135	168-174	1
29	13	99-99	121-121	122-122	135-135	174-174	1
30	143	99-99	121-121	131-135	135-135	168-174	1
31	10, 79, 141	99-99	121-121	134-135	138-144	168-168	3
32	69	99-99	121-121	134-135	138-145	168-168	1
33	50	99-99	121-121	135-135	135-135	168-174	1
34	144	99-99	121-121	135-135	138-144	168-168	1
35	67	99-99	121-121	135-138	135-135	168-168	1
36	24	99-99	121-121	135-138	135-135	168-171	1
37	56	99-99	121-121	135-138	135-135	168-174	1
38	106, 172, 173	99-99	121-121	135-144	135-135	168-174	3
39	138, 151	99-99	121-121	135-144	138-138	168-168	2
40	22, 23, 41, 43, 94, 122, 135, 155	99-99	121-121	135-145	135-135	168-168	8
41	2, 3, 48, 53, 55, 59, 60, 61, 66, 71, 75, 80, 83, 88, 93, 108, 113, 142, 160	99-99	121-121	135-145	135-135	168-174	19
42	97	99-99	121-121	135-145	135-135	168-180	1
43	37	99-99	121-121	135-145	135-135	169-174	1
44	12, 44, 51, 52, 81, 158	99-99	121-121	135-145	135-135	174-174	6
45	139, 140, 157	99-99	121-121	135-145	135-138	168-168	3
46	58, 72, 73	99-99	121-121	135-145	135-138	168-174	3
47	116	99-99	121-121	135-145	135-138	174-174	1
48	34	99-99	121-121	135-145	135-138	180-180	1
49	161	99-99	121-121	135-149	135-135	168-168	1
50	91	99-99	121-121	135-149	135-135	168-174	1
51	128, 137	99-99	121-121	145-145	135-135	168-168	2
52	28, 86, 120, 159	99-99	121-121	145-145	135-135	168-174	4
53	109	99-99	121-121	145-145	135-135	174-174	1
54	104	99-99	121-121	145-145	135-138	162-174	1
55	164	99-99	121-121	145-145	135-138	168-174	1
56	168	99-99	121-127	144-144	135-138	168-180	1
57	42	99-99	121-127	145-145	135-135	168-174	1
58	29, 114, 156	99-101	118-121	130-130	135-135	171-180	3
59	54, 162	99-101	121-121	130-130	135-135	171-180	2
60	107	99-101	127-127	131-135	138-138	168-168	1
61	82	99-104	115-118	131-135	138-144	168-168	1
62	46, 63	99-104	115-118	132-144	138-145	168-168	2
63	98	99-104	115-118	132-144	145-145	168-168	1
64	62	99-104	118-118	134-135	135-135	168-168	1
65	64	99-104	118-118	135-149	138-141	171-180	1
66	89	99-104	119-121	134-135	135-144	168-171	1
67	8, 110	99-104	121-121	134-135	135-138	168-168	2
68	112	99-107	118-118	119-129	138-138	168-168	1
69	132	99-107	118-118	135-144	135-138	168-180	1
70	131	99-107	118-118	135-144	138-138	168-180	1
71	171	99-107	118-118	135-149	135-138	168-180	1
72	87	99-107	118-118	144-144	138-138	168-180	1
73	20	99-107	118-121	122-122	145-145	168-171	1
74	147	99-107	118-121	135-145	135-144	168-168	1
75	16, 21	99-107	118-130	122-122	138-138	168-180	2
76	33	99-107	118-130	134-145	138-138	168-180	1
77	27, 117	99-107	118-130	135-144	138-138	168-168	2
78	92, 96, 115, 154, 166	99-107	118-130	135-144	138-138	168-180	5
79	11	99-107	118-130	135-149	138-138	168-168	1
80	134	99-107	119-130	135-144	138-138	168-180	1
81	26	99-107	119-130	135-149	138-138	168-168	1
82	170	99-107	119-130	135-149	138-138	180-180	1
83	103	99-107	130-130	135-135	138-138	168-180	1
84	39, 49	99-107	130-130	135-144	138-138	168-180	2
85	15	99-112	118-121	120-120	135-144	168-168	1
86	17, 77	99-112	118-121	124-134	135-145	168-168	2
87	78	99-112	118-130	134-135	138-144	171-171	1
88	153	99-112	119-119	124-134	135-135	168-168	1
89	100	99-112	119-130	134-135	138-144	168-171	1
90	123	101-101	118-121	130-130	135-135	171-180	1
91	165	101-101	119-121	130-130	135-135	171-180	1
92	145	101-101	121-121	129-135	135-138	168-168	1
93	99	101-101	121-127	129-129	135-138	168-168	1
94	30, 124, 136	101-101	121-127	129-135	135-138	168-168	3
95	174	104-107	118-121	135-135	135-138	168-168	1
96	146	104-107	118-130	129-135	135-135	168-171	1
97	119	104-107	118-130	129-135	135-138	168-168	1
98	74, 85, 95, 102, 129, 133, 149, 163	104-107	118-130	129-144	135-138	168-171	8
99	127	104-107	118-130	129-153	135-138	168-168	1
100	40	104-107	119-130	129-144	135-138	168-168	1
101	9	104-107	119-130	129-144	135-138	168-171	1
102	125	104-112	121-121	134-135	138-145	168-174	1
103	68, 70	107-107	118-118	129-144	135-138	168-171	2
104	126	107-107	118-118	130-130	135-138	168-168	1

## Discussion

*Candida* species account for a substantial proportion of healthcare-associated bloodstream infections. Understanding the genotypic diversity of these pathogens, along with their antifungal resistance profiles, is essential for developing strategies to prevent and control such infections. This study examined the genetic diversity and antifungal susceptibility of a large collection of bloodstream *C. albicans* strains. No association was found between antifungal susceptibility and the clinical source of the isolates.

Consistent with previous reports [2, 4, 10, 16, 17], the tested azole antifungals exhibited strong *in vitro* activity against all *C. albicans* strains, with only a single isolate showing resistance to fluconazole. Resistance to amphotericin B and flucytosine was minimal. Results of this study also align with those of earlier studies, indicating that echinocandin resistance in *C. albicans* is rare [18–21].

Data on the genetic diversity and antifungal susceptibility of *C. albicans* in the Eastern Mediterranean Region remain limited. Recently, Dagi et al. [10] evaluated the antifungal susceptibility of six antifungal agents against 95 *C. albicans* bloodstream isolates from Konya, Turkey, and found that all strains were susceptible to amphotericin B, with no resistance detected to voriconazole, posaconazole, caspofungin, or anidulafungin.

Similarly, in a 5-year *C. albicans*-resistance surveillance at a Brazilian university hospital [22], all blood isolates were classified as susceptible to all evaluated antifungal agents, with MICs ranges of 0.125-1.00 mg/L for amphotericin B, ≤ 0.125-1.00 mg/L for flucytosine, ≤ 0.125-0.5 mg/L for fluconazole, ≤ 0.015-0.125 mg/L for itraconazole, ≤ 0.015-0.06 μg/mL for voriconazole, and ≤ 0.015-0.125 mg/L for caspofungin. In another study performed by Taj-Aldeen et al. [23], resistance to amphotericin B, itraconazole, fluconazole, voriconazole, anidulafungin, caspofungin, posaconazole, and isavuconazole was not detected in any of the *C. albicans* strains isolated from candidemia patients in Qatar.

As presented in Tables 2 and 3, the results revealed considerable genetic diversity among *C. albicans* isolates causing candidemia, based on allelic variations across five microsatellite loci (CAIII, CEF3, KRE6, LOC4, and ZNF1). In a locus-by-locus analysis, the largest number of allelic genotypes was observed with CEF3, which enabled the identification of 21 genotypes, followed by CAIII with 12 clusters.

Escribano et al. [8] used a microsatellite panel of six short-tandem repeats to analyze the genetic diversity of 217 *C. albicans* isolates from the blood cultures of 202 patients with candidemia. They observed identical genotypes across all six markers in some isolates, while 174 distinct genotypes were identified overall, including 155 unique and 19 endemic genotypes confined to the same hospital unit.

In another study conducted by Dalle et al. [7], 15 distinct CEF3 profiles were reported among bloodstream strains of *C. albicans*. Additionally, genotypes 126–135, 130–136, and 131–131 comprised 60.4% of both bloodstream and control strains [7]. The authors of the present study previously investigated a collection of 216 vaginal *C. albicans* isolates and identified 20 allelic combination-based genotypes using microsatellite marker analysis of CEF3, wherein genotypes 17 (136–144) and 2 (126–135) accounted for 50% of the isolates [11].

The present study used the combination of 5 loci and demonstrated a combined DP of 0.98. These loci were selected due to their reported high DP [12]. To achieve higher resolution among different genotypes, previous studies used more than two microsatellite markers for *C. albicans* genotyping [24–26]. Although microsatellite genotyping is a rapid and highly reproducible method that allows detection of different alleles at a given locus to distinguish heterozygotes in diploid organisms, such as *C. albicans* [25, 27, 28], other molecular typing techniques, including pulsed-field gel electrophoresis [29], multilocus sequence typing [24, 30], restriction fragment length polymorphism [31], and randomly amplified polymorphic DNA [32], have also been successfully used to differentiate *C. albicans* isolates.

It is important to note that before typing of *Candida* isolates, accurate species identification should be performed using either PCR-based sequencing of target genes or commercial proteomic approaches, such as matrix-assisted laser desorption/ionization time-of-flight mass spectrometry.

A limitation of this study was its single-center design in Izmir, Turkey, which may restrict the generalizability of the findings to other regions or healthcare settings. Additionally, the use of only five microsatellite markers, while providing high discriminatory power, may not capture the full extent of genetic diversity, compared to more comprehensive genotyping methods. This study also lacked detailed patient clinical data, which could have helped correlate genotypic profiles with clinical outcomes. Finally, environmental and epidemiological investigations were not performed to confirm potential sources of infection within the hospital.

## Conclusion

In conclusion, this study revealed that antifungal resistance was extremely rare among bloodstream *C. albicans* isolates in Izmir. The investigated genotypes displayed considerable diversity, and there were no statistical relationships between the patterns of susceptibility to each antifungal compound and the different genotypes studied. However, the presence of identical microsatellite genotypes among some strains suggests a potential common source of infection within the hospital, warranting further investigation.

This study provides valuable insights into the genotypic diversity of bloodstream-derived *Candida* isolates and their antifungal susceptibility profiles. These findings may support the development of preventive strategies to better control nosocomial *Candida* infections.
